# Viruses in the Oceanic Basement

**DOI:** 10.1128/mBio.02129-16

**Published:** 2017-03-07

**Authors:** Olivia D. Nigro, Sean P. Jungbluth, Huei-Ting Lin, Chih-Chiang Hsieh, Jaclyn A. Miranda, Christopher R. Schvarcz, Michael S. Rappé, Grieg F. Steward

**Affiliations:** a Department of Oceanography, University of Hawai'i at Mānoa, Honolulu, Hawai'i, USA; b Hawai'i Institute of Marine Biology, University of Hawai'i at Mānoa, Kāne'ohe, Hawai'i, USA; Oregon State University

## Abstract

Microbial life has been detected well into the igneous crust of the seafloor (i.e., the oceanic basement), but there have been no reports confirming the presence of viruses in this habitat. To detect and characterize an ocean basement virome, geothermally heated fluid samples (ca. 60 to 65°C) were collected from 117 to 292 m deep into the ocean basement using seafloor observatories installed in two boreholes (Integrated Ocean Drilling Program [IODP] U1362A and U1362B) drilled in the eastern sediment-covered flank of the Juan de Fuca Ridge. Concentrations of virus-like particles in the fluid samples were on the order of 0.2 × 10^5^ to 2 × 10^5^ ml^−1^ (*n* = 8), higher than prokaryote-like cells in the same samples by a factor of 9 on average (range, 1.5 to 27). Electron microscopy revealed diverse viral morphotypes similar to those of viruses known to infect bacteria and thermophilic archaea. An analysis of virus-like sequences in basement microbial metagenomes suggests that those from archaeon-infecting viruses were the most common (63 to 80%). Complete genomes of a putative archaeon-infecting virus and a prophage within an archaeal scaffold were identified among the assembled sequences, and sequence analysis suggests that they represent lineages divergent from known thermophilic viruses. Of the clustered regularly interspaced short palindromic repeat (CRISPR)-containing scaffolds in the metagenomes for which a taxonomy could be inferred (163 out of 737), 51 to 55% appeared to be archaeal and 45 to 49% appeared to be bacterial. These results imply that the warmed, highly altered fluids in deeply buried ocean basement harbor a distinct assemblage of novel viruses, including many that infect archaea, and that these viruses are active participants in the ecology of the basement microbiome.

## IMPORTANCE

The hydrothermally active ocean basement is voluminous and likely provided conditions critical to the origins of life, but the microbiology of this vast habitat is not well understood. Viruses in particular, although integral to the origins, evolution, and ecology of all life on earth, have never been documented in basement fluids. This report provides the first estimate of free virus particles (virions) within fluids circulating through the extrusive basalt of the seafloor and describes the morphological and genetic signatures of basement viruses. These data push the known geographical limits of the virosphere deep into the ocean basement and point to a wealth of novel viral diversity, exploration of which could shed light on the early evolution of viruses.

## INTRODUCTION

The first viruses appear to have arisen very early in the history of life on earth (1) and have been coevolving with cells ever since. Viruses infect every known type of organism, and they appear to be a ubiquitous feature of all biological communities. They have been documented in nearly every habitat where life has been found (2–4), including deeply buried marine sediments (5–7) and in fluids emanating from submarine hydrothermal vents (8–10). However, one major habitat for which no evidence of viruses or viral infections has yet been recorded is the igneous ocean crust (10).

Hydrothermal vents have been described as a window into the conditions and processes occurring deeper in the basement (11), and it is possible that some of the viruses previously observed in vent fluid samples originated from deeper in the igneous crust. However, vent fluids are subject to contamination from seawater entrainment and local recirculation through the chimneys and surface sediments. The provenance of the viruses observed in samples of vent fluids is therefore uncertain, and the types and concentrations of the viruses reported likely do not accurately represent those deeper in the basement. As a consequence, the roughly 20 million km^3^ of fluids percolating through the oceanic basement (12) constitutes an enormous ecosystem for which we know nothing about the resident viral assemblages.

Although viruses have not yet been reported in the ocean basement, there is convincing evidence of prokaryotic life there (13). Some of this evidence has come from examination of rock cores, but small sample sizes, low biomass, and the difficulty of avoiding contamination pose analytical challenges (14). An alternative approach to study the basement habitat is to sample the fluids that circulate through the basement rather than the rock itself (70). This became feasible with the development and installation of seafloor observatories called CORKs (circulation obviation retrofit kits), which are placed into existing boreholes (15). With recent improvements in the CORK design (16) and with CORK-compatible *in situ* sampling equipment (17), it is now possible to sample up to hundreds of liters of pristine basement fluids for microbiological analysis.

**FIG 1:**
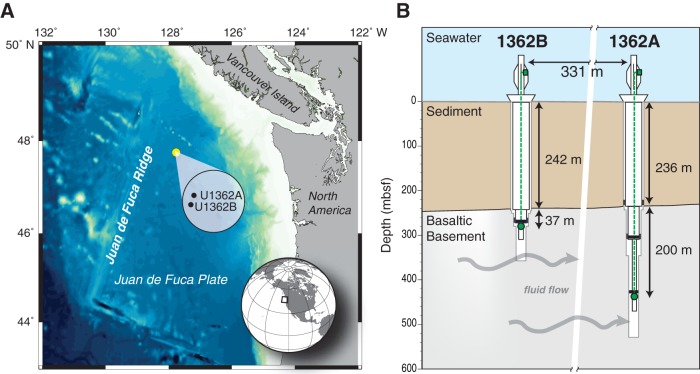
Location and schematic of the Juan de Fuca Ridge CORKs U1362A and U1362B. (A) Map illustrating the location (yellow dot) and relative orientation (expanded circle) of IODP boreholes U1362A and U1362B on the eastern flank of the Juan de Fuca Ridge. (B) Schematic of CORKs U1362A and U1362B illustrating the depths of the holes and the sampling intakes relative to the sediment and basement horizons. Titanium screen sampling inlet (green circle) is connected by PTFE line to a valved sampling port (green square) on the seafloor wellhead. Packers (black rectangles) just above each inlet isolate the lowest depth horizon in each hole. Depth (in meters below the seafloor [mbsf]) is shown on the *y* axis.

Two recent CORKs, U1362A and U1362B, have been installed into 3.5 million-year-old basaltic crust on the Juan de Fuca Ridge (JdFR) flank in the northeastern Pacific Ocean (18). These two CORKs penetrate hundreds of meters below the seafloor first through sediment and then through 292 (U1362A) or 117 (U1362B) m of basalt (Fig. 1), allowing access to basalt-hosted crustal fluids. These basement fluids originated as bottom seawater, which was entrained at unsedimented and lightly sedimented ridges and distant outcrops. The fluids have been geothermally warmed to ca. 65°C and chemically altered as a result of interactions with the crust during transit through cracks and fissures in the basalt (19). Previous chemical analyses of fluid samples from U1362A and U1362B revealed that the basement fluid at these sites is near neutral (pH 7.3 to 7.5) with an alkalinity of 0.5 to 0.6 (milliequivalents per liter) (20). Dissolved organic carbon (DOC) in the samples (11 to 16 µM) was low compared to bottom seawater (40 to 45 µM), and both oxygen and nitrate were exhausted from the fluids, leaving sulfate as the likely dominant electron acceptor (20, 21). Methane (CH_4_) and hydrogen (H_2_) were present in nano- to micromolar concentrations within basement fluids and likely serve, along with residual DOC, as electron donors (21).

JdFR basement fluids contain a much lower concentration of microbial cells and a distinct community composition compared to overlying sediments and seawater, as inferred through metagenomics, genomes amplified from single cells, and small-subunit rRNA (SSU rRNA) gene amplicon sequencing surveys (20, 22). A limited number of bacteria and archaea appear to dominate this system, including lineages within candidate bacterial phyla Aminicenantes, Acetothermia, EM3, and Calescamantes, candidate archaeal phyla marine benthic group E (MBG-E), Bathyarchaeota, and the terrestrial hot spring Crenarchaeotic group (THSCG), and divergent lineages only distantly related to characterized cultivars within the phyla *Nitrospirae*, *Chloroflexi*, and *Archaeoglobi*. The presence of distinct microbial assemblages inhabiting this system is consistent with the unique nature of the crustal fluids within this subseafloor basement aquifer.

On the basis of this prior evidence of distinct microbial communities in the basement fluids of the JdFR flank, we expected that a distinct assemblage of viruses would be present in these fluids as well. To test this, we leveraged over a decade of technological developments to analyze extra- and intracellular viruses in pristine, CORK-derived samples. We used epifluorescence microscopy to determine the concentrations of extracellular virions in basement fluids at these sites and electron microscopy to observe their morphological types. We then analyzed metagenomes prepared from the microbial size fraction (>0.2 µm) at the same two locations to identify and characterize virus-like sequences associated with the cell fraction. Finally, we analyzed sequence scaffolds containing clustered regularly interspaced short palindromic repeat (CRISPR) elements (23) to determine what types of cells in the deep subseafloor harbor this type of microbial immune system and are thus likely to have been subjected to recent infections.

## RESULTS

### Abundance and morphology of extracellular virions.

**FIG 2:**
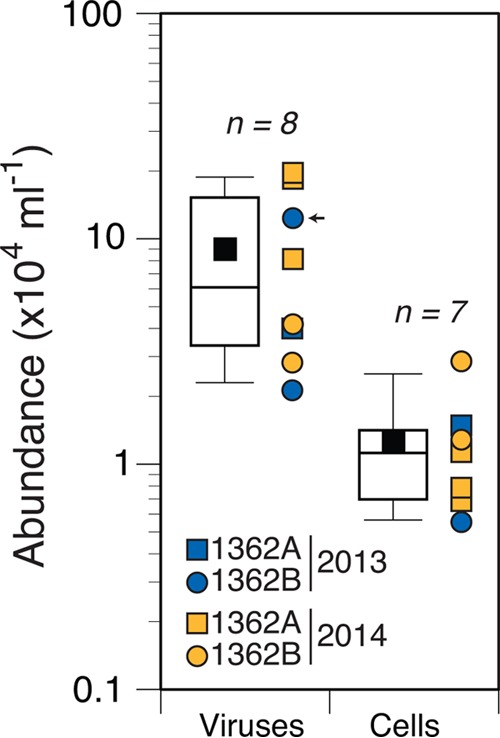
Concentrations of putative viruses (*n* = 8) and prokaryotic cells (*n* = 7) in basaltic basement fluids as determined by staining characteristics using epifluorescence microscopy. For each category, box-and-whisker plots are shown to the left and depict the mean (black square) and the 10th, 25th, 50th, 75th, and 90th percentiles of counts. The individual counts from 2013 (blue symbols) or 2014 (orange symbols) for CORK U1362A (squares) or U1362B (circles) are shown to the right of the box-and-whisker plots. In one instance (indicated by a small black arrow), only a filtered sample (<0.2 µm) was available, so only viruses were counted.

**FIG 3:**
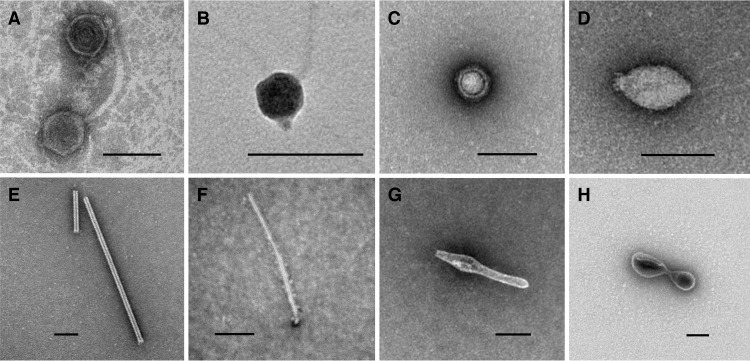
Morphologies of viruses observed in basalt-hosted crustal fluid. (A to H) Electron micrographs of particles harvested from borehole U1362A revealed tailed viruses similar to members of the order *Caudovirales* (A and B), untailed viruses (C), lemon-shaped viruses resembling members of the family *Bicaudaviridae* or *Fuselloviridae* (D), rod-shaped viruses resembling members of the family *Rudiviridae* (E), and other particles resembling filamentous (F) or spindle-shaped (G) viruses isolated from *Archaea*. Bilobate structures in the viral size range (H) were repeatedly observed, but they were unlike any classified viruses. These might represent a novel virus or may be membrane vesicles. Bars, 100 nm.

Chemical analysis of bag sample fluids indicated that the purity of the samples collected for epifluorescence microscopy (EfM) ranged from 99 to 100% (Table 1). The average concentration of viruses (SYBR gold-stained virus-like particles) in these samples was 9 × 10^4^ ml^−1^ (standard deviation [SD], 7 × 10^4 ^ml^−1^; range, 2 × 10^4 ^to 20 × 10^4^ ml^−1^; *n* = 8), and the average concentration of cells (SYBR gold-stained prokaryote-like bodies) was 1 × 10^4^ ml^−1^ (SD, 0.8 × 10^4 ^ml^−1^; range, 0.5 × 10^4^ to 3 × 10^4^ ml^−1^; *n* = 7) (Fig. 2). The variability in counts within a single sampling event (standard deviation expressed as a percentage of the mean) was 41 to 57% for viruses, and it was 25 to 65% for cells. The overall variability for the entire data set was 78 and 62% for viruses and cells, respectively. Eukaryote-like cells were not detected, but an exhaustive search was not performed. With 60 to 400 cells enumerated per sample, the detection limit was roughly 1.6 to 0.24% of the community. The virus-to-cell ratio varied from 1.5 to 27 (average, 9; *n* = 7). Bottom seawater near the CORK contained 3 × 10^6^ viruses ml^−1^ and 6 × 10^4^ cells ml^−1^ with a virus-to-cell ratio of 17 (*n* = 1). Qualitative examination of purified viruses in the borehole fluid samples from U1362A by transmission electron microscopy (TEM) revealed tailed and untailed icosahedral, untailed globular, and lemon-, rod-, or spindle-shaped morphotypes (Fig. 3).

**TABLE 1:** Summary of analyses performed in this study and characteristics of samples^a^

Analysis^b^	Sample date (yr-mo-day)	Location^c^	Dive no.^d^	Collection method^e^	Mg^2+^ concn (mM)	Crustal fluid content (%)	Concn (×10^4^ ml^−1^)	
							Viruses	Cells
Meta	2011-07-10	U1362B	J2-571	*In situ* F	NA	NA	NA	NA
	2011-07-12	U1362A	J2-573	*In situ* F	NA	NA	NA	NA
EfM	2013-07-14	U1362B	J2-710	MVBS	2.3–2.4	100	12.4^f^	0.0^f^
	2013-07-15	U1362A	J2-711	MVBS	2.5–3.1	99–100	3.9	1.5
	2013-07-21	U1362B	J2-715	LVBS	2.4	100	2.1	0.54
	2014-08-15	U1362A	AL-4754	LVBS	2.2	100	18	0.67
	2014-08-22	U1362A	AL-4760	LVBS	2.5	99	8.1	0.78
	2014-08-22	U1362A	AL-4760	MVBS	2.6	99	19	1.1
	2014-08-09	U1362B	AL-4757	MVBS	2.2	100	2.8	1.3
	2014-08-09	U1362B	AL-4757	LVBS	2.1	100	4.1	2.8
	2014-08-11	nbSW	NA	Niskin	53.0	0	110	6.2
TEM	2014-8-15 to2014-08-21	U1362A	AL-4754 toAL4759	*In situ* UF	NA	NA	NA	NA

a Summary of analyses performed in this study, the dates and sources of the samples for each analysis, the Mg^2+^ concentration in bag-collected samples, the calculated purity of those samples (percent crustal fluid content), and the concentrations of viruses and cells in the bag samples as estimated by epifluorescence microscopy. NA, not available.

Analyses are microbial metagenomics (Meta), epifluorescence microscopy (EfM), and transmission electron microscopy (TEM).

c Locations are referred to by the CORK number or near-bottom seawater (nbSW).

d Dives from the ROV *Jason II* are indicated by a J2 prefix, and dives from the HOV *Alvin* are indicated by an AL prefix.

e The samples were collected by a large- or medium-volume bag sampler (LVBS or MVBS), *in situ* direct 0.22-µm filtration (*in situ* F), *in situ* direct 100-kDa ultrafiltration (*in situ* UF), or collection by using a Niskin bottle (Niskin).

f Sample counts are for filtrate of basement fluid (<0.22-µm filter [Sterivex; Millipore]).

### Domains represented in the microbial metagenomes of basement fluids.

Analysis of SSU rRNA genes in the assembled reads of microbial metagenomes prepared from basement fluids collected at U1362A and U1362B indicated that bacterial SSU rRNA genes dominated the communities at both sites (67% and 76%, respectively). Archaeal SSU rRNA genes were also common (33% and 23%, respectively), but those of eukaryotes were rare (0% and 1%). When considering the phylogenetic assignment for all predicted genes, archaea represented a higher proportion of the total (44% in U1362A and 34% in U1362B).

### Putative hosts for viruses in the microbial metagenomes.

**FIG 4:**
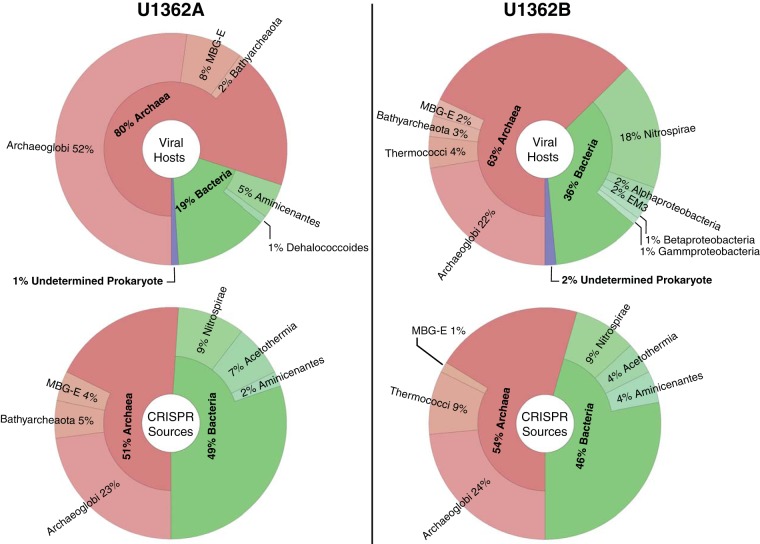
Taxonomic affiliations of the inferred hosts for the virus-like scaffolds identified by VirSorter (top) and of the CRISPR-containing scaffolds (bottom) in the assembled metagenomes (>0.2-µm fraction) prepared from basement fluid samples retrieved from U1362A or U1362B. MBG-E, marine benthic group E.

A total of 150 out of 137,575 total scaffolds (0.1%) and 154 out of 212,307 total scaffolds (0.07%) in U1362A and U1362B, respectively, were identified by VirSorter as likely deriving from viruses. After normalizing for scaffold read depth, the majority of all of the virus-affiliated reads (80% in U1362A and 63% in U1362B) were inferred to have archaeal hosts based on the taxonomy of the genomic bin to which a scaffold was assigned or the consensus taxonomic assignments of BLASTP hits (nr database) of genes on each scaffold (Fig. 4).

Of the VirSorter-identified scaffolds placed in archaeal genomic bins, most were classified as *Archaeoglobi*, with smaller proportions (<10%) of Bathyarchaeota, marine benthic group E (MBG-E), or *Thermococci* (U1362B only). Of the VirSorter-identified reads from U1362A and U1362B inferred to be from viruses having a bacterial host (19 and 36%, respectively), the largest identified group was *Nitrospirae* (18% in U1362B only) with smaller contributions (≤5%) from Aminicenantes, *Chloroflexi* (U1362A), uncharacterized bacterial group EM3 (U1362B), and various proteobacteria. Another 1 to 2% of the reads were inferred to be from viruses infecting prokaryotes, but with no consensus assignment as bacterial or archaeal.

Approximately 19% (U1362A) and 14% (U1362B) of the VirSorter-identified scaffolds were specifically identified as likely prophages. For those cases where a prophage host assignment could be inferred based both on BLASTP (nr) analysis and genomic bin affiliation, the agreement between the two methods was 97% (U1362A; *n* = 28) and 100% (U1362B; *n* = 21). In cases of disagreement, the genomic bin affiliations were used to infer the likely host. The percentage of archaeal genomic bins containing prophages (73%) was more than twice as high as the percentage of bacterial bins (34%).

### Inferred taxonomy of CRISPR-containing scaffolds.

CRISPR arrays were detected in 0.30% (U1362A) and 0.16% (U1362B) of the total scaffolds. The taxonomic affiliation of most of these CRISPR-containing scaffolds in each sample could not be reliably classified (76% and 80% in U1362A and U1362B, respectively), because they were too short or had too few genes with significant similarity to genes from known sources. For the scaffolds that were assigned to a domain, those annotated as archaeal accounted for 51% (U1362A) and 54% (U1362B), with the remainder annotated as bacterial (Fig. 4). The CRISPR-containing lineages specifically identified were mostly the same as those identified as likely hosts for the viruses detected in the microbial metagenome. One notable difference was the identification of 7% (U1362A) and 4% (U1362B) of the CRISPR-containing scaffolds as deriving from candidate phylum Acetothermia (OP1). This group was not identified as the likely host for any of the virus-like scaffolds identified by VirSorter.

### Taxonomic similarities with known viruses.

**FIG 5:**
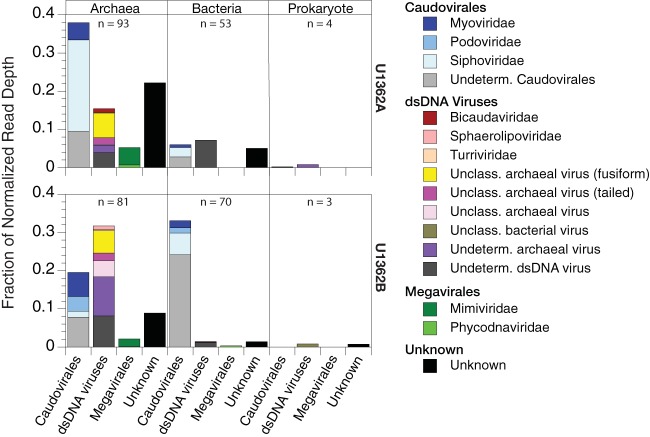
Consensus taxonomic affiliation of the best BLASTP hits for the genes on the virus-like scaffolds identified by VirSorter in metagenomes from U1362A or U1362B using only viral sequences from RefSeq as the target database and clustered by the most likely domain for the putative host of the virus. The data are presented as the fraction of scaffolds classified in each category after normalizing for read depth, but the absolute number of scaffolds (*n*) is also shown for each host category. “Unclassified (Unclass.)” indicates significant similarity (E value of 10^−5^) to specific viruses that are not yet classified in the NCBI taxonomy database (as of 2 September 2016). “Undetermined (Undeterm.)” indicates that there was no consensus in the taxonomy among the significant BLAST hits beyond the level of order. “Unknown” classifications indicate no significant hits to the viral RefSeq database. An exception to the NCBI-based taxonomy is the inclusion of *Megavirales*, which is currently a proposed, but not yet official, order (69).

The closest taxonomic affiliations for each virus-like scaffold inferred from the top BLAST matches to the viral RefSeq database suggested that many scaffolds from putative archaeal viruses had one or more genes most similar to those from diverse archaeal viruses that have not yet been classified (Fig. 5). Of the scaffolds with highest similarity to classified viruses, most of the matches were to tailed viruses. Specifically, similarities were detected to members of the families *Myoviridae* and *Siphoviridae* in the order *Caudovirales*, as well as to morphologically similar tailed archaeal viruses that have not yet been officially classified (all haloviruses). Also common were scaffolds with similarity to viruses with a known fusiform morphology (some bicaudavirids, but most unclassified). For some of the putative archaeal virus scaffolds, the only genes on the scaffold with significant similarity to any known virus were most similar to genes from viruses in the proposed order *Megavirales*. To date, this order includes only viruses that infect eukaryotes.

The majority of putative bacterial virus scaffolds from U1362B had genes most similar to members of the *Caudovirales*. Although some showed consensus among hits to a particular family (*Myo*-, *Podo*-, or *Siphoviridae*), most showed no consensus among the hits at the family level. Similarities to members of the *Caudovirales* were also detected among scaffolds from U1362A, but most bacterial virus-like scaffolds were not resolved beyond having similarity to double-stranded DNA (dsDNA) viruses.

### Analysis of complete viral genomes.

**FIG 6:**
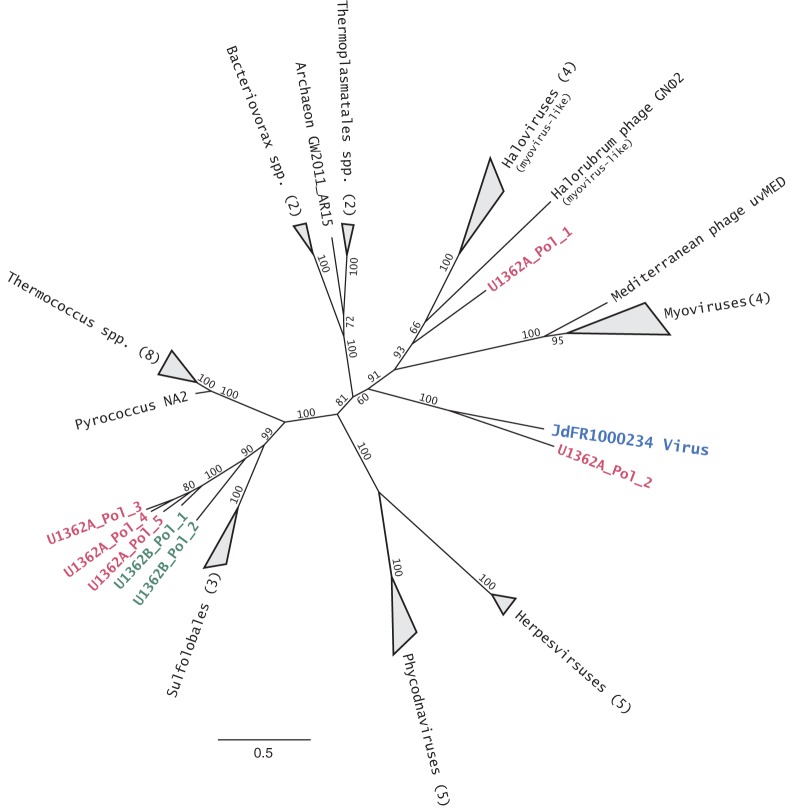
Maximum-likelihood phylogeny of the DNA polymerase II gene (*polB*) from viral genome JdFR1000234 (in blue boldface font) and homologous viral and archaeal amino acid sequences. Values at the nodes indicate percent bootstrap support, and the bar indicates 0.05 substitutions per site. Bootstrap values at some internal nodes were omitted for clarity. Colored font indicates sequences that were obtained from the U1362A (red) or the U1362B (green) metagenomes. A cluster of the sequences derived from these metagenomes branch within archaeal *polB* genes and are likely derived from archaea. The DNA polymerase B gene from JdFR1000234 forms a monophyletic lineage with another sequence from borehole U1362A that is different from all other known *polB* sequences. Identification numbers for the sequences are listed in Table S3.

One of the scaffolds identified by VirSorter (JdFR1000234) appears to be a complete, circular viral genome or circularly permuted viral genome. The genome is 55,906 nucleotides (nt) long and has a G+C content of 35.2%. Of the 81 identified open reading frames (ORFs), 20 were annotated with a putative function (see Table S1 in the supplemental material); none were tRNAs. The three predicted proteins with the most significant BLAST-based similarities (all BLAST E values of <10^−30^) were to distinctively viral proteins (major capsid, portal, and terminase) of archaeal haloviruses that have a myovirus-like morphology. Another predicted protein had a domain with a significant match to an archaeal/eukaryotic core primase in the conserved domain database (PriL; cl11970). Phylogenetic analysis of a putative DNA polymerase B gene indicated that it is highly divergent from other homologous genes in known viruses or cells (Fig. 6). The closest relative to the JdFR1000234 sequence is a DNA polymerase B sequence derived from the U1362A microbial metagenome that is part of a large, incomplete viral scaffold (JGI scaffold identification [ID] number JGI24020J35080_1000327). The nearest characterized viruses are myoviruses and myovirus-like haloviruses.

10.1128/mBio.02129-16.1 TABLE S1  Gene annotations for virus-like genome JdFR1000234. Download TABLE S1, PDF file, 0.04 MB. Copyright © 2017 Nigro et al. 2017 Nigro et al. This content is distributed under the terms of the Creative Commons Attribution 4.0 International license.

Another VirSorter-identified scaffold (JdFRA1000001) appears to be a complete prophage genome containing 42 ORFs, of which only 7 are similar to proteins in the databases searched here. The 27,660-bp prophage spans from nt 116,961 to 144,621 of the 540,961-nt scaffold. Although no tRNAs were found within the prophage, a sequence identified as tRNA- Tyr(GTA) (Integrated Microbial Genomes [IMG] gene ID JGI24020J35080_1000001181) is located adjacent to the terminal integrase gene of the prophage. The phenomenon of integration of prokaryotic mobile elements adjacent to tRNAs is well described (24) and supports the identification of this genomic feature as a prophage. The G+C content of the prophage is 39.3%, while the G+C content of the remaining fragment is similar at 40.6%. The predicted proteins include an integrase, helicase, recombinase, and protease (Table S2). No genes encoding a DNA polymerase or other common viral structural components such as head or tail proteins were identified by BLAST-based similarity searches. The microbial portion of the scaffold was classified both by characterization of its full-length SSU rRNA gene and phylogenomic analysis of the parent genome bin that includes this scaffold (25); both indicate that the host of this virus is a novel lineage of the terrestrial hot springs Crenarchaeotic group (THSCG) within the archaeal phylum *Aigarchaeota*.

10.1128/mBio.02129-16.2 TABLE S2  Gene annotations for putative prophage JdFRA1000001. Download TABLE S2, PDF file, 0.03 MB. Copyright © 2017 Nigro et al. 2017 Nigro et al. This content is distributed under the terms of the Creative Commons Attribution 4.0 International license.

## DISCUSSION

The data reported here provide the first direct evidence that viruses are present deep in the ocean basement, and considering the physical context and nature of the assemblage, it is most likely that these viruses are produced there as a result of infections of an indigenous basement microbiota. The basement at the study site is covered in a thick layer of sediment (~240 m), which prevents local convective exchange with the overlying seawater. The nearest identified recharge site, Grizzly Bare seamount (26), is nearly 60 km away (27). Based on modeled flow through the basement and the highly altered chemistry and elevated temperature, the fluid at the sampling site is believed to have entered the basement as bottom seawater decades to centuries ago (19). Our analyses of the chemistry of collected fluid samples imply that the CORKs delivered pristine basement fluids with little to no contamination from bottom seawater, and because of the high temperature and age of the fluids and the distinctiveness of the viral assemblage, it is highly unlikely that these viruses are simply slow-decaying vestiges of viruses from the source seawater. Our data suggest instead that they are likely active participants in the ecology of the basement microbiome.

### Viral abundance and morphology.

Our estimates of the abundance of prokaryotes are consistent with previous reports on prokaryotes in JdFR basement fluids (20, 22) but now include the first corresponding estimates of viruses. The estimates of viral concentrations in the basalt-hosted fluids were 1 to 2 orders of magnitude lower than those in overlying bottom seawater at this location and lower than those in fluids emanating from hydrothermal vents (8, 9, 28). The higher values in the latter studies are likely a consequence of anoxic vent fluids mixing with the oxygenated bottom seawater driving high, localized chemosynthetic production (29).

Although our data help to constrain the concentration of viruses in the deep basement habitat on the JdFR flank, our counts are restricted to the fluid phase. We do not know whether the cells and viruses in the harvested fluids represent a distinct free-living community or whether they are derived from similar populations of surface-attached microorganisms. In either case, previous observations of surface-attached microorganisms in basement basalts (30) suggest that the effective concentrations of cells and viruses in the basement could be greater than our counts indicate. Epifluorescence microscopy has other limitations as a method for detecting viruses. Some viruses with small genomes, for example, may not be detected (31), and some portion of the virus-like particles could be vesicles derived from cells (32, 33).

More convincing evidence of the presence of viruses in the oceanic basement is provided by electron microscopy, which revealed a suite of diverse morphologies. Many of the viruses resembled those that infect thermophilic archaea (34), an observation consistent with the elevated temperatures at the study site as well as the detection of presumed archaeal thermophiles through DNA sequencing (20, 25). Similar lemon- and rod-shaped morphologies have been observed in high-temperature enrichment cultures inoculated with pieces of hydrothermal vent chimney or surrounding sediments (35). Other viruses resembled the tailed viruses that comprise the order *Caudovirales*, which are among the more common morphologies in other types of marine habitats, such as seawater (36) and sediments. Although most of the officially classified members of the order *Caudovirales* infect bacteria, similar morphologies are common among euryarchaeon-infecting haloviruses that have yet to be classified (37).

### Viruses in the microbial metagenomes.

Our intent in analyzing the viral signal in metagenomes of the nominal cellular fraction (>0.2 µm) for this study was specifically to supplement the evidence of free virions in the basement fluids with evidence that viruses are interacting with the basement microbes. The identification of prophages indicates that there are persistent infections of cells by temperate viruses, and the identification of CRISPR elements indicates that infections are frequent enough to select for maintenance of a microbial immune system in those microbes. Viral sequences can also be detected in metagenomes from the >0.2-µm fraction for other reasons, including adsorption of free virions onto the filter, viral particles that are larger than the filter pore size, or the presence of intracellular virions accumulating during lytic infections. For these reasons, metagenomes prepared from fractions targeting cells can provide insights into the viral community (9, 38–40). The analysis of virus-like sequences in the two metagenomes confirmed the qualitative results from the morphological analysis and suggested that the basement harbors a diverse assemblage of viruses infecting both archaea and bacteria, many of which are highly divergent from known viruses. Although some scaffolds had genes that were most similar to those of eukaryotic viruses in the proposed order *Megavirales*, analysis of the scaffolds suggested that, in most cases, the likely hosts were not eukaryotes, but archaea. This may reflect an evolutionary relationship between some archaeal viruses and megavirads, as was proposed earlier based on analysis of the replication machinery (41).

The prokaryotic community assemblage found in the deep subsurface of the JdFR is unique; however, many of the major lineages have relatives found in marine and terrestrial hydrothermal environments. This includes, for example, lineages related to the archaeal genera *Archaeoglobus* and *Methanococcus* and the bacterial genera *Thermotoga* and *Aquifex*. The JdFR crustal fluids, in general, harbor a microbial assemblage consistent with a system that is experiencing high temperatures, based on the temperature optima of the closest known relatives (20).

The prevalence of viruses resembling thermophilic archaeal viruses in the CORK metagenomes is consistent with the evidence that thermophilic archaea constitute a significant fraction of the microbial community in the warm anoxic crustal fluids at the study site (20), but it is unusual among viromes from the marine habitats analyzed thus far. This is true even of hydrothermal vent metagenomes (42) where one might expect them to be common. Furthermore, there have not been any prior reports of the corresponding lemon-, spindle-, or rod-shaped viral morphologies being directly observed in any type of marine samples (pelagic or benthic), except for an instance of a lemon-shaped virus that was isolated following selective enrichment (35). The prevalence of such viruses in our basement samples probably reflects the nature of the sampled habitat, which has elevated temperatures over large horizontal expanses. Venting sites, in contrast, have fluctuating and steep gradients in temperature and chemistry over very short spatial scales, with corresponding variations in microbial communities (43). Previous studies from such sites may have simply missed the thermophilic archaea and their viruses because the surrounding populations of nonthermophilic microorganisms may easily overwhelm them.

Supporting the evidence from scaffold classifications that archaeal viruses were common, two complete viral genomes assembled from the library appear to be derived from viruses infecting archaea. The majority of the best BLAST hits to the complete circularized genome of virus JdFR1000234, including the three most significant matches, were to archaeal viruses, and the putative primase most closely resembled the archaeal/eukaryotic type rather than the bacterial/bacteriophage type. While these gene similarities and the DNA polymerase-based phylogeny suggest that the genome derives from an archaeal virus, it is highly divergent, so the specific phylogenetic affiliation of the host is not certain. The reconstruction of the same complete genome at both sampling sites suggests that it was relatively common in the two microbial metagenomes. If the reconstructed genome is derived from a phage with a contractile tail, as implied by the phylogenetic analysis, preferential retention of long-tailed viruses on 0.2-µm-pore-size filters (44) could have contributed to the observed relative enrichment.

A more specific archaeal host affiliation was inferred for the prophage JdFRA1000001 that resides within a scaffold assigned to a novel archaeal lineage of the THSCG. Integration by crenarchaeal viruses has been previously observed (37), and like JdFRA1000001, other crenarchaeal viruses have few ORFs (45). It is not uncommon for their genomes to be lacking homologs to DNA polymerase genes or to head and tail proteins. Some crenarchaeal viruses do not share any genes with other known viruses (46). Morphologically, these viruses are fundamentally different from typical bacteriophages (i.e., *Caudovirales*) and euryarchaeal viruses with caudovirad-like head-tail morphologies (47), so our observation that the putative proteins of this virus show little similarity to other known viral proteins is not surprising.

Also noteworthy in the metagenomes was the low percentage of sequences annotated as eukaryotic, which suggests that eukaryotes are relatively rare in the hot, highly altered basement fluids of the JdFR flank. Viruses, rather than protistan predators, therefore may be the most important source of mortality for bacteria and archaea in this basement habitat. The identification of CRISPR elements in a high percentage of both bacterial and archaeal scaffolds provides additional evidence that invasion of cells by viruses or other mobile elements is common and is expected to influence the ecology and evolution of the basement microbiome.

In conclusion, our results provide the first documentation of the abundance, morphology, and phylogenetic affiliation of viruses in the highly altered, basalt-hosted fluids of oceanic crust. This work greatly expands the known geographical range of viruses from ocean sediments to deep within sediment-covered seafloor basalt. The novel lineages observed in just this one location, together with the known large variation in physical and chemical conditions within the ocean basement globally (48), implies that the deep subsurface harbors a much greater wealth of viral diversity than that uncovered so far. The data imply that viral processes are important in the ecology of the deep subsurface, and suggest that the ocean basement is a promising habitat in which to search for deeply branching viral lineages that could shed light on the early origins and diversification of cells and their associated viruses.

## MATERIALS AND METHODS

### Sample site.

Basement fluid samples were collected in July 2011, July 2013, and August 2014 during R/V *Atlantis* cruises AT18-07, AT26-03, and AT26-18 using the ROV *Jason II* (AT18-07 and AT26-03) or the submersible HOV *Alvin* (AT26-18) (Table 1). Fluid samples were obtained from Integrated Ocean Drilling Program (IODP) boreholes U1362A (47°45.6628′N, 127°45.6720′W) and U1362B (47°45.4997′N, 127°45.7312′W), which are located at an ocean depth of ~2,650 m on the eastern flank of the Juan de Fuca Ridge (JdFR) in the northeastern Pacific Ocean (27). The holes penetrate through ~240 m of sediment and then extend another 292 m (U1362A) or 117 m (U1362B) into underlying basaltic basement for total hole depths of 528 or 359 m below the seafloor (mbsf), respectively (Fig. 1). The holes are equipped with CORKs (circulation obviation retrofit kits) that allow for sample retrieval from predetermined depth horizons using valve-controlled ports at a wellhead on the seafloor (49). Armored fluid delivery lines (1.27-cm inner diameter) of polytetrafluoroethylene (PTFE) extend from the wellhead ports to 436 mbsf (U1362A) or 279 mbsf (U1362B), which is 200 or 37 m subbasement (msb), respectively, and terminate in titanium microscreens secured on the outside of a coated casing. Inflatable and swellable packers located approximately 10 m above the sample inlets isolate the deepest depth interval in each hole (193 to 292 msb for U1362A and 30 to 117 msb for U1362B).

### Sample collection.

Fluid-tight connection of sampling equipment via umbilicals to the wellheads and manipulation of valves was achieved using the robotic arms of the ROV or HOV. Most samples were collected with a custom-built system that allows for active *in situ* pumping of fluids directly through filters or into acid-washed polyvinyl fluoride bags for shipboard processing (17). One sample was collected by passive *in situ* filtration using only the pressure differential between formation fluid and bottom seawater. In all cases, valves were used to purge sample lines (three times the estimated volume of the CORK fluid delivery lines and sampling umbilicals) prior to collection.

In 2011, basement fluids intended for metagenome sequencing were collected by pumping fluids through 0.22-µm-pore-size polyethersulfone filter cartridges (Steripak-GP20; Millipore, Billerica, MA, USA) *in situ* from U1362A (124 liters) and U1362B (70 liters). In 2013 and 2014, samples for microscopy were collected from U1362A and U1362B on multiple occasions by active pumping into bags for shipboard processing. In 2014, a large volume of fluids from U1362A was filtered *in situ* through a 0.2-µm-pore-size capsule filter (Polycap 75; Whatman), and an ultrafiltration filter unit with a 100-kDa nominal-molecular-weight-cutoff (NMWCO) membrane (Pellicon 2 [2 m^2^]; EMD Millipore) connected in series. The entire fluid path was filled with virus-free water (Viresolve NFP; Millipore) and sealed prior to deployment. Using only the positive pressure from the basement formation fluids, ~10,000 liters of deep subsurface fluids passed through the filter during a 6-day deployment. For comparison with basement fluid virus and cell counts, a sample of near-bottom (2,646 m) seawater was collected by using Niskin bottles in 2014.

### Determining sample integrity.

For bag-collected fluid samples, subsamples were analyzed by ion chromatography to determine Mg^2+^ concentrations to test for contamination of the highly altered subsurface fluids with unaltered seawater. The purity of the sample was inferred from a two end-member mixing model as previously described (21).

### Epifluorescence microscopy.

Bag samples (whole or 0.2 µm filtered in one instance) for analysis by epifluorescence microscopy (EfM) were subsampled and fixed with 2% formaldehyde (0.02 µm filtered) in 2013 or 1.9% paraformaldehyde and 0.96% glutaraldehyde (electron microscopy grade) in 2014. Samples for EfM were frozen and stored at −80°C (2013) or flash frozen in liquid nitrogen (2014) and stored at −80°C immediately after shipboard sample recovery. To prepare slides, preserved basement fluid samples were thawed, and the samples (25 to 75 ml) were passed through a 0.02-µm aluminum oxide filter (Anodisc; GE Healthcare). Filters were poststained from below with SYBR gold as previously described (50). Smaller, more dimly stained particles were categorized as viruses. Larger, brighter particles with cell-like morphologies (e.g., coccoid, bacilloid, vibrioid, and filamentous) were categorized as cells. The concentrations of particles were estimated using an epifluorescence microscope (Nikon i90) directly or from digital photographs by counting 20 different view fields in all but one case (five fields counted).

### Transmission electron microscopy (TEM).

Viruses could not be eluted from the *in situ* ultrafilter until after the cruise so they were preserved as suggested by Steward and Culley (51) by pumping 1 liter of 0.02-µm-filtered RNALater (Thermo Fisher Scientific, Waltham, MA) into the inlet to displace the residual sample fluid. The high ammonium sulfate concentration in RNALater is bacteriostatic (52), but it preserves the integrity and even the infectivity of viruses (53–55). The filter was stored in the housing at 4°C for 1 month until the viruses could be eluted. Viruses were recovered from the ultrafilter by repeated back flushing and simultaneous tangential flow recirculation with SM buffer (100 mM NaCl, 8 mM MgSO_4_, 50 mM Tris [pH 7.5]) supplemented with Tween 80 (0.05%) to aid in desorption of viruses from the membrane (56). After each of three rounds of back flushing, the recovered fluid samples were concentrated by centrifugal ultrafiltration (Centricon 80 [30-kDa NMWCO]; Millipore) to a few milliliters, and then all final concentrates were pooled. Viruses were purified by two rounds of centrifugation in continuous CsCl gradients (57). Fractions were collected using a piston fractionator (Gradient Station; Biocomp Instruments) and then exchanged into SM buffer by centrifugal ultrafiltration. Subsamples of the gradient-purified viruses were preserved with 2% electron microscopy-grade paraformaldehyde and then centrifuged at 53,000 × *g* for 15 min, followed by 90,700 × *g* for 30 min in an EM-90 rotor in an Airfuge (Beckman Coulter, Inc.) to deposit viruses onto carbon-stabilized Formvar supports on 200-mesh copper grids (Ted Pella). Material on the supports was imaged in a transmission electron microscope (HT7700; Hitachi) at 100 keV accelerating voltage, and digital images were captured digitally (AMT XR-41).

### Metagenome preparation.

In 2011, following *in situ* filtration and retrieval of the Steripak-GP20 filters, lysis buffer (20 mM Tris-HCl, 2 mM EDTA, 1.2% Triton X-100, 2% [wt/vol] lysozyme [pH 8]) was added to the filter capsules, the ends were sealed, and the units were stored at −80°C. DNA was extracted from filters using a phenol-chloroform extraction protocol (20). As described in detail elsewhere (25), metagenome sequencing of the resulting environmental DNA was performed by the Joint Genome Institute (JGI) using an Illumina HiSeq-2000 platform, resulting in 170 million and 168 million sequence reads from boreholes U1362A and U1362B, respectively. Sequences were processed following standard JGI protocols for metagenomes, which included quality filtering, followed by assembly and annotation. Standard operating procedures for metagenome processing and annotation performed at JGI can be found at https://img.jgi.doe.gov/m/doc/MetagenomeAnnotationSOP.pdf (58). The resulting data are available at the JGI Integrated Microbial Genomes (IMG) web portal with IMG genome IDs of 3300002481 (U1362A) and 3300002532 (U1362B).

### Identification and classification of viral sequences.

Putative viral sequences were retrieved from the metagenome assemblies using the program VirSorter v1.0.3 (59) with default parameters through the iPlant Collaborative Discovery Environment on 25 September 2015. Both the RefSeqABVir and Virome databases were used, which allowed for categorization of both free-living viruses and prophages. Scaffolds predicted by VirSorter at all three confidence levels to be derived from viruses or to contain prophages were used for subsequent analyses. All of the predicted protein-coding regions on each of the identified scaffolds were extracted, translated, and used as queries to identify similar sequences via BLASTP (v2.4.0+) (60) within either the RefSeq viral database (downloaded 29 June 2016) or the NCBI nr database (downloaded 4 April 2016).

Inferences about the most likely viral taxonomic affiliation (family level or above) for a scaffold were based on consensus among BLAST hits (E value of ≤10^−5^) to the RefSeq viral database for all proteins encoded on a given scaffold. The number of proteins encoded on an individual scaffold with hits to this database ranged from 1 to 39. If there was a single, significant BLASTP hit (true for 20 to 27% of the scaffolds), the affiliation of the matching virus was used. If there were two or more proteins encoded on a scaffold with significant hits to viruses, taxonomic assignment was to the lowest level for which there was consensus among the hits. The consensus assignment was that for which both the two hits with the lowest E values and the majority of all hits were in agreement.

### Inferring taxonomic affiliation of viral hosts and CRISPR-containing scaffolds.

Inferences about the likely hosts of the VirSorter-identified viruses were based on one of two criteria. The first was the presence of the scaffold in a genomic bin. Ninety-eight genomic bins were established for the U1362A and U1362B metagenomic libraries as described elsewhere (25). If a VirSorter-identified scaffold was assigned to one of those bins, then taxonomic assignment of that bin (to the lowest level identified) was taken as the likely host. In cases where a scaffold was not placed in a genomic bin, then the likely host was inferred (to the domain level only) based on the consensus taxonomic affiliation among BLASTP hits to the NCBI nr database for all proteins encoded on the scaffold. In cases where the best BLASTP hit was to a virus, the domain of the host for that virus was used. Consensus criteria were as stated above for assignment of viral taxonomy.

CRISPR elements were identified on assembled scaffolds by IMG with CRT (61) and PILER-CR (62). Taxonomic affiliation of the CRISPR-containing scaffolds was inferred in the same manner as above with one modification. In the cases where scaffolds were not assigned to genomic bins, host identity was inferred based on JGI-assigned BLAST-based identifications, but only on scaffolds at least 2,500 bp in length and with at least three annotated genes. For scaffolds not meeting these criteria, the taxonomic affiliation was classified as unknown.

### Analysis of complete viral genomes.

Two scaffolds, JGI24020J35080_1000234 and JGI24019J35510_1000079, from metagenomic data sets U1362A and U1362B, respectively, were determined by VirSorter to contain complete, circularized genomes. Alignment of these genomes using Geneious v6.1.6 (63) revealed that the two genomes are 100% identical. This genome sequence is hereafter referred to as JdFR1000234. Coding sequences within the genome were predicted using the MetaGeneAnnotater (MGA) (64), as well as using the IMG DOE-JGI Metagenome Annotation Pipeline (MAP) (58). Final coding sequence determinations were made manually. Functional annotations were made by identifying homologous proteins from the Conserved Domain Database (CDD), as well as using InterProScan to search the ProDom, PRINTS, PIR-PSD, PSD, SMART, TIGRFAM, PROSITE, and SUPERFAMILY databases. The program tRNAscan-SE was used to search for tRNAs (65).

A phylogenetic tree of the DNA polymerase B gene (pfam00136) from JdFR1000234 and relatives was constructed. Using the MaFFT program (v7.017) (66) with default parameters, an amino acid alignment was created with the JdFR1000234 DNA polymerase B gene and homologous sequences obtained through BLASTP searching of both the NCBI nr and the viral subset (taxon 10239) of the NCBI nr database and against the U1362A and U1362B metagenome assemblies via the IMG online portal. A list of taxon names and sequence ID numbers are listed in Table S3 in the supplemental material. From this alignment, a maximum likelihood tree was created using the PhyML 3.0 program, with the SPR topology search method (67). The tree was bootstrapped 1,000 times (Fig. 6).

10.1128/mBio.02129-16.3 TABLE S3  Identification numbers for sequences used in maximum-likelihood tree (Fig. 6). Download TABLE S3, PDF file, 0.04 MB. Copyright © 2017 Nigro et al. 2017 Nigro et al. This content is distributed under the terms of the Creative Commons Attribution 4.0 International license.

VirSorter also identified a complete prophage genome from the U1362A metagenome assembly (JGI scaffold ID JGI24020J35080_1000001), which is on a fragment that also contains a complete SSU rRNA gene. The microbial portion of the scaffold was classified both by characterization of its full-length SSU rRNA gene, using the Silva online classifier (68) and phylogenomic analysis of the parent genome bin that includes this scaffold (25). This viral portion of this scaffold, named prophage JdFRA1000001, was extracted and annotated in the same manner as described above.

### Accession number(s).

Annotations of JdFR1000234 and JdFRA1000001 have been submitted to GenBank and assigned accession numbers KY229235 and KY229234, respectively. Metagenomes from U1362A and U1362B are available at the JGI Integrated Microbial Genomes (IMG) web portal using the IMG genome IDs 3300002481 and 3300002532, respectively.
