# Supplementary material for: Viruses in the Oceanic Basement
Source: mBio. 2017 Mar 7;8(2):e02129-16. doi: 10.1128/mBio.02129-16 (PMC5340873; doi:10.1128/mBio.02129-16)
Supplement: TABLE S1 [file mbo001173218st1.pdf]

Table S1. Gene annotations for virus-like genome scaffold JdFR1000234

SeqName	Description	Coordinates	Coding Direction
JdFR1000234_gene_01	Archaeal/Eukaryotic type DNA primase large subunit	1..949	+
JdFR1000234_gene_02	DNA polymerase	924..2936	+
JdFR1000234_gene_03	---NA---	2963..3208	+
JdFR1000234_gene_04	---NA---	3208..3864	+
JdFR1000234_gene_05	5' to 3' Cas4-like exonuclease	3914..4558	+
JdFR1000234_gene_06	---NA---	4691..5098	+
JdFR1000234_gene_07	DUF1599-containing protein	5095..5532	+
JdFR1000234_gene_08	---NA---	5525..5683	+
JdFR1000234_gene_09	---NA---	5733..5891	+
JdFR1000234_gene_10	Site-specific DNA-methyltransferase	5895..6716	+
JdFR1000234_gene_11	ERCC4 nuclease	6707..7414	+
JdFR1000234_gene_12	---NA---	7386..7772	+
JdFR1000234_gene_13	Terminase large subunit	7772..9130	+
JdFR1000234_gene_14	---NA---	9097..9372	+
JdFR1000234_gene_15	---NA---	9799..10233	+
JdFR1000234_gene_16	---NA---	10349..10558	+
JdFR1000234_gene_17	Acetyltransferase	10530..10850	+
JdFR1000234_gene_18	Ribosomal protein S30	10935..11150	+
JdFR1000234_gene_19	---NA---	11143..11292	+
JdFR1000234_gene_20	---NA---	11294..11464	+
JdFR1000234_gene_21	---NA---	11487..11882	+
JdFR1000234_gene_22	---NA---	11900..12076	+
JdFR1000234_gene_23	---NA---	12078..12242	+
JdFR1000234_gene_24	---NA---	12248..12934	+
JdFR1000234_gene_25	---NA---	12941..13348	+
JdFR1000234_gene_26	---NA---	14013..14264	+
JdFR1000234_gene_27	---NA---	14265..14507	+
JdFR1000234_gene_28	---NA---	14512..14835	+
JdFR1000234_gene_29	---NA---	14918..15058	+
JdFR1000234_gene_30	---NA---	15059..15388	+
JdFR1000234_gene_31	---NA---	15385..15585	+
JdFR1000234_gene_32	---NA---	15784..16086	+
JdFR1000234_gene_33	---NA---	16049..16291	+
JdFR1000234_gene_34	Acyltransferase	16401..18905	+
JdFR1000234_gene_35	Glutamine amidotransferase class 2	18910..20322	+
JdFR1000234_gene_36	---NA---	20319..20783	+
JdFR1000234_gene_37	---NA---	20767..20892	+
JdFR1000234_gene_38	---NA---	20876..21031	+
JdFR1000234_gene_39	---NA---	21037..21183	+
JdFR1000234_gene_40	---NA---	21437..21757	+

JdFR1000234_gene_41	---NA---	21763..22047	+
JdFR1000234_gene_42	---NA---	22044..22247	+
JdFR1000234_gene_43	---NA---	22247..22591	+
JdFR1000234_gene_44	---NA---	22604..22924	+
JdFR1000234_gene_45	---NA---	22927..23331	+
JdFR1000234_gene_46	ParB-like nuclease domain containing protein	23328..25259	+
JdFR1000234_gene_47	---NA---	25259..25567	+
JdFR1000234_gene_48	AAA ATPase	25578..26699	+
JdFR1000234_gene_49	Putative metal-dependent peptidase	26686..27897	+
JdFR1000234_gene_50	---NA---	27894..28559	+
JdFR1000234_gene_51	---NA---	28642..29265	+
JdFR1000234_gene_52	---NA---	29243..29671	+
JdFR1000234_gene_53	---NA---	29658..30260	+
JdFR1000234_gene_54	---NA---	30272..30400	+
JdFR1000234_gene_55	---NA---	30405..31325	+
JdFR1000234_gene_56	---NA---	31380..31721	+
JdFR1000234_gene_57	---NA---	31724..31942	+
JdFR1000234_gene_58	---NA---	31915..33372	+
JdFR1000234_gene_59	---NA---	33383..33793	+
JdFR1000234_gene_60	Hypothetical protein containing DUF2341	33796..38904	+
JdFR1000234_gene_61	---NA---	39049..39405	+
JdFR1000234_gene_62	Laminin G domain-containing protein	39407..40801	+
JdFR1000234_gene_63	---NA---	40803..41924	+
JdFR1000234_gene_64	---NA---	41936..42154	+
JdFR1000234_gene_65	---NA---	42169..42351	+
JdFR1000234_gene_66	---NA---	42404..42736	+
JdFR1000234_gene_67	---NA---	42759..43553	+
JdFR1000234_gene_68	---NA---	43562..43963	+
JdFR1000234_gene_69	Phage portal protein	43947..45707	+
JdFR1000234_gene_70	---NA---	45712..47379	+
JdFR1000234_gene_71	---NA---	47354..48754	+
JdFR1000234_gene_72	---NA---	48742..48930	+
JdFR1000234_gene_73	Phage-like element PBSX protein	48974..50476	+
JdFR1000234_gene_74	---NA---	50427..51263	+
JdFR1000234_gene_75	Putative major capsid protein	51269..52426	+
JdFR1000234_gene_76	---NA---	52468..52737	+
JdFR1000234_gene_77	---NA---	52805..53428	+
JdFR1000234_gene_78	---NA---	53474..53692	+
JdFR1000234_gene_79	---NA---	53695..54465	+
JdFR1000234_gene_80	---NA---	54462..54833	+
JdFR1000234_gene_81	Putative archaeal type recombinase	54958..55875	+
