# Supplementary material for: Viruses in the Oceanic Basement
Source: mBio. 2017 Mar 7;8(2):e02129-16. doi: 10.1128/mBio.02129-16 (PMC5340873; doi:10.1128/mBio.02129-16)
Supplement: TABLE S2 [file mbo001173218st2.pdf]

Table S2. Gene annotations for putative prophage scaffold JdFRA1000001

SeqName	Description	Coordinates	Coding Direction
JdFRA1000001_gene_01	Putative 7-cyano-7-deazaguanine tRNA-ribosyltransferase	49..1597	+
JdFRA1000001_gene_02	---NA---	1608..1982	+
JdFRA1000001_gene_03	---NA---	2375..2677	+
JdFRA1000001_gene_04	---NA---	2824..3828	+
JdFRA1000001_gene_05	---NA---	4127..4330	-
JdFRA1000001_gene_06	---NA---	4428..5240	-
JdFRA1000001_gene_07	---NA---	5475..5684	-
JdFRA1000001_gene_08	---NA---	5789..6298	-
JdFRA1000001_gene_09	Serine Recombinase	6855..7454	-
JdFRA1000001_gene_10	---NA---	7451..7621	-
JdFRA1000001_gene_11	---NA---	7599..8513	-
JdFRA1000001_gene_12	---NA---	8564..8908	-
JdFRA1000001_gene_13	---NA---	8905..9198	-
JdFRA1000001_gene_14	---NA---	9205..9576	-
JdFRA1000001_gene_15	---NA---	9676..10248	-
JdFRA1000001_gene_16	Serine Protease	10245..11048	-
JdFRA1000001_gene_17	---NA---	11029..11460	-
JdFRA1000001_gene_18	---NA---	11465..11803	-
JdFRA1000001_gene_19	---NA---	11793..12962	-
JdFRA1000001_gene_20	---NA---	12963..14723	-
JdFRA1000001_gene_21	---NA---	14726..15364	-
JdFRA1000001_gene_22	---NA---	15398..16807	-
JdFRA1000001_gene_23	---NA---	17044..17448	+
JdFRA1000001_gene_24	---NA---	17471..17734	+
JdFRA1000001_gene_25	---NA---	17731..18174	+
JdFRA1000001_gene_26	---NA---	18191..18418	-
JdFRA1000001_gene_27	Superfamily II DNA or RNA helicase	18424..19953	-
JdFRA1000001_gene_28	---NA---	19993..20448	-
JdFRA1000001_gene_29	Phage protein Gp37/Gp68	20748..21434	-
JdFRA1000001_gene_30	---NA---	21427..21843	-
JdFRA1000001_gene_31	---NA---	21922..22938	-
JdFRA1000001_gene_32	---NA---	22954..23340	-
JdFRA1000001_gene_33	---NA---	23337..23597	-
JdFRA1000001_gene_34	---NA---	23594..23794	-
JdFRA1000001_gene_35	---NA---	23795..24118	-
JdFRA1000001_gene_36	---NA---	24233..24601	-
JdFRA1000001_gene_37	---NA---	24719..24871	-
JdFRA1000001_gene_38	---NA---	24864..25268	-
JdFRA1000001_gene_39	---NA---	25381..25644	+
JdFRA1000001_gene_40	---NA---	25630..25803	-
JdFRA1000001_gene_41	Prefoldin	25940..26530	+
JdFRA1000001_gene_42	Integrase	26536..27660	-
