# Supplementary material for: Viruses in the Oceanic Basement
Source: mBio. 2017 Mar 7;8(2):e02129-16. doi: 10.1128/mBio.02129-16 (PMC5340873; doi:10.1128/mBio.02129-16)
Supplement: TABLE S3 [file mbo001173218st3.pdf]

Table S3. Identification numbers for sequences used in maximum likelihood tree

Condensed Clade ID	Taxon Name	Genbank Accession Number	IMG Genome ID	IMG Scaffold ID	IMG Gene ID
	archaeon GW2011_AR15	AJF61662.1			
	Halorubrum phage GNΦ2†	AGF91223.1			
	JdFR1000234			Ga0100361_1000234	
	Mediterranean phage uvMED (uncult.)	BAR37019.1			
	Pyrococcus sp. NA2	WP_013748330.1			
	U1362A Pol 1			JGI24020J35080_1001695	
	U1362A Pol 2			JGI24020J35080_1000327	
	U1362A Pol 3			JGI24020J35080_1000024	
	U1362A Pol 4			JGI24020J35080_1000135	
	U1362A Pol 5			JGI24020J35080_1001689	
	U1362B Pol 1			JGI24019J35510_1000741	
	U1362B Pol 2			JGI24019J35510_1000071	
Bacteriovorax	Bacteriovorax marinus		2563366500		2563369050
	Bacteriovorax sp. BAL6_X		2545824765		2546729032
Haloviruses*	Halovirus HF1	NP_542554.1			
	Halovirus HRTV-7	YP_008060026.1			
	Halovirus HRTV-8	YP_008058620.1			
	Halovirus HSTV-2	YP_007379123.1			
Herpesviruses	Gorilla herpesvirus 7	AIN81096.1			
	Human herpesvirus 7	AGV28662.1			
	Human herpesvirus 7	YP_073778.1			
	Macaca nemestrina herpesvirus 7	YP_009253942.1			
	Pan troglodytes herpesvirus 7	AIN81099.1			
Myoviruses	Cyanophage P-TIM40	YP_009188250.1			
	Pelagibacter phage HTVC008M	YP_007517987.1			
	Prochlorococcus phage P-SSM4	YP_214707.1			
	Synechococcus phage S-SM2	YP_004322306.1			
Phycodnaviruses	Micromonas pusilla virus 12T	YP_007676285.1			
	Ostreococcus lucimarinus virus 7	YP_009173227.1			
	Ostreococcus mediterraneus virus 1	YP_009172985.1			
	Ostreococcus tauri virus 2	YP_004063640.1			
	Yellowstone lake phycodnavirus 1	YP_009174732.1			
Sulfolobales	Metallosphaera sedula	WP_012022080.1			
	Metallosphaera yellowstonensis	WP_048088122.1			
	Sulfolobales archaeon		2524614637		2525231854
Thermococcus	pGDR11-Tgo	ALL53335.1			
	Thermococcus celer	ADK60920.1			
	Thermococcus gammatolerans EJ3	WP_015859068.1			
	Thermococcus gorgonarius	P56689.1			
	Thermococcus gorgonarius	2VWJ_A			
	Thermococcus gorgonarius	2XHB_A			
	Thermococcus peptonophilus	WP_062388189.1			
	V93q Polymerase Variant	2VWK_A			
Thermoplasmatales	Thermoplasmatales archaeon DG-70	KYK38881.1			
	Thermoplasmatales archaeon DG-70-1	KYK36290.1			

† myovirus-like morphology (Shereen Sabet; personal communication)

* myovirus-like morphologies (reviewed by Senčilo and Roine, 2014. Front. Microbiol. 5: 84; doi: 10.3389/fmicb.2014.00084)
